# Supplementary material for: Cell Adhesion Molecule 1 Contributes to Cell Survival in Crowded Epithelial Monolayers
Source: Int J Mol Sci. 2020 Jun 9;21(11):4123. doi: 10.3390/ijms21114123 (PMC7312920; doi:10.3390/ijms21114123)
Supplement: Supplementary file 1 [file ijms-21-04123-s001.pdf]

Supplementary materials: Cell Adhesion Molecule 1 Contributes to Cell Survival in Crowded Epithelial Monolayers

Man Hagiyama, Ryuichiro Kimura, Azusa Yoneshige, Takao Inoue, Tomoyuki Otani and Akihiko Ito

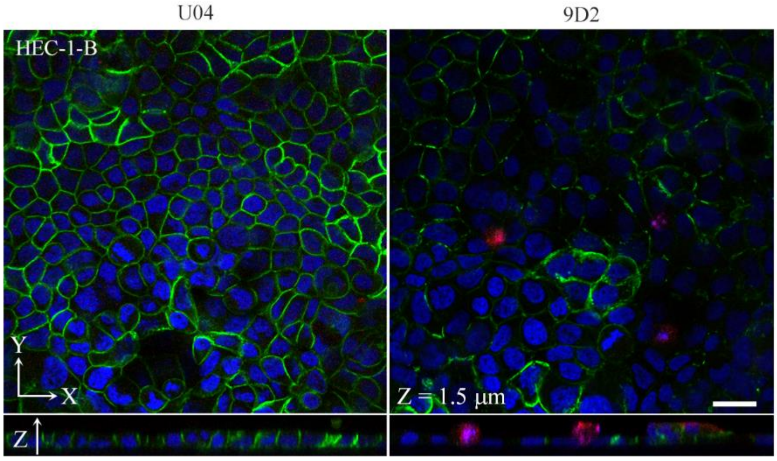

Figure S1. 9D2 induces apoptosis in crowded HEC-1-B cells and decreases the cell height. HEC-1-B cells were cultured on a semipermeable membrane in 12-well plates. When the cells reached 100% confluence, control IgY U04 or 9D2 was added at a concentration of 10 μg/ml. After 2 days, the cells were triple-stained with CADM1 immunofluorescence (3E1 antibody; green), TUNEL method (red), and DAPI nuclear staining (blue). HEC-1-B cells treated with 9D2 were micrographed in an X-Y plane at the Z axis of about 1.5 μm. Note that the number of TUNEL-positive cells are smaller than that in Figure 5, which X-Y plane is at the Z axis of about 3.5 μm. Scale bar = 20 μm.

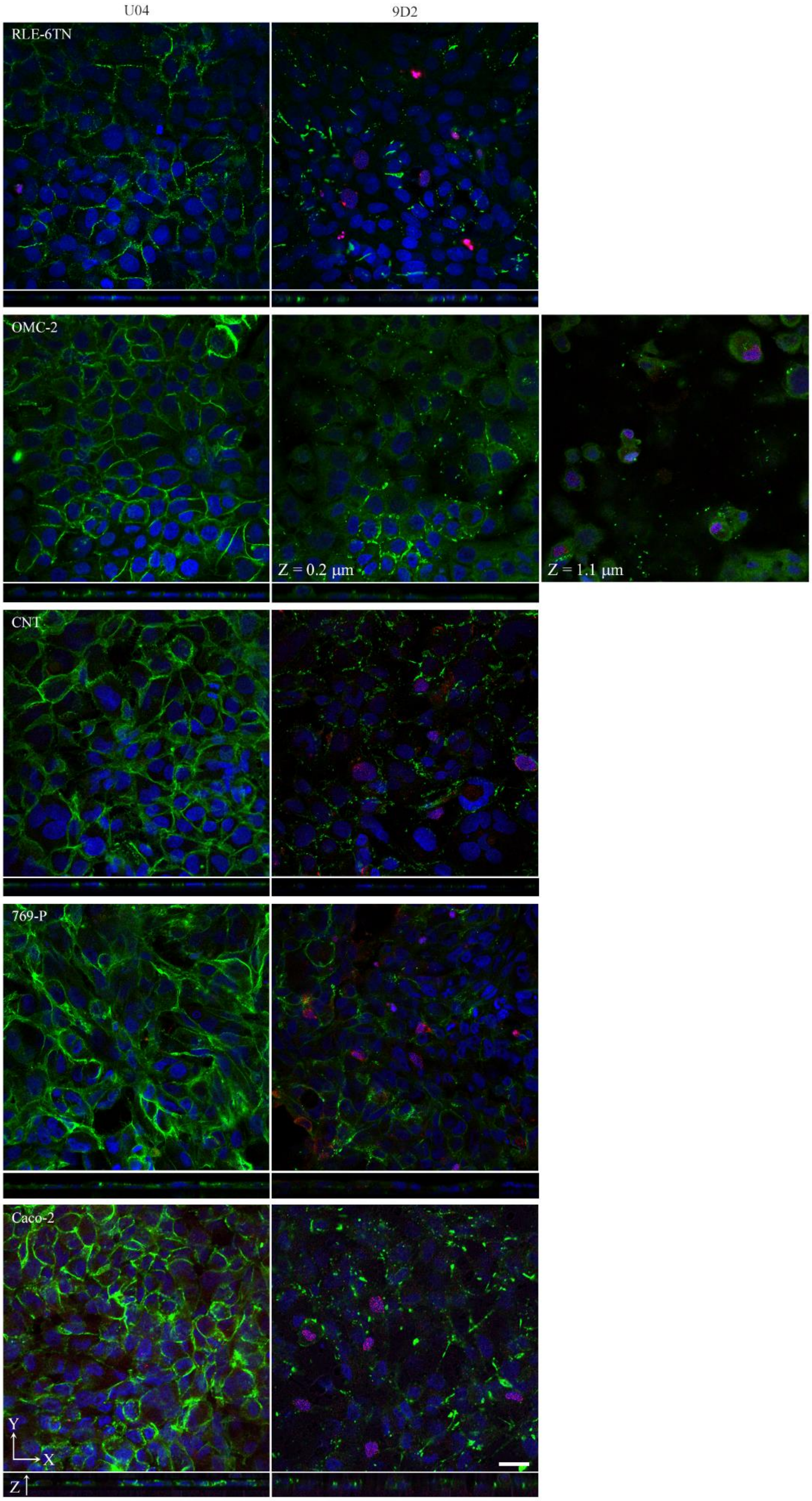

Figure S2. 9D2 induces apoptosis in crowded epithelial cell monolayers. Various cell lines indicated were cultured on a semipermeable membrane in 12-well plates. When the cells reached 100% confluence, control IgY U04 or 9D2 was added at a concentration of 10 μg/ml. After 2 days, the cells

were triple-stained with CADM1 immunofluorescence (3E1 antibody; green), TUNEL method (red), and DAPI nuclear staining (blue). OMC-2 cells treated with 9D2 were micrographed in two X-Y planes at the Z axis of 0.2 and 1.1 μm. Note that the number of TUNEL-positive cells are larger in the X-Y plane at Z = 1.1 μm. Scale bar = 20 μm.

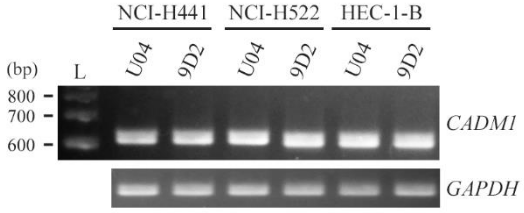

Figure S3. Reverse transcription-PCR analyses for the *CADM1* mRNA. Three cell lines indicated were cultured on a semipermeable membrane in 12-well plates. When the cells reached 100% confluence, control IgY U04 or 9D2 was added at a concentration of 10 μg/ml. After 2 days, total RNA was extracted from the cells, reverse transcribed, and was PCR-amplified using a primer set for *CADM1* or glyceraldehyde 3-phosphate dehydrogenase (GAPDH). The PCR products were electrophoresed on agarose gels. L, ladder size marker.

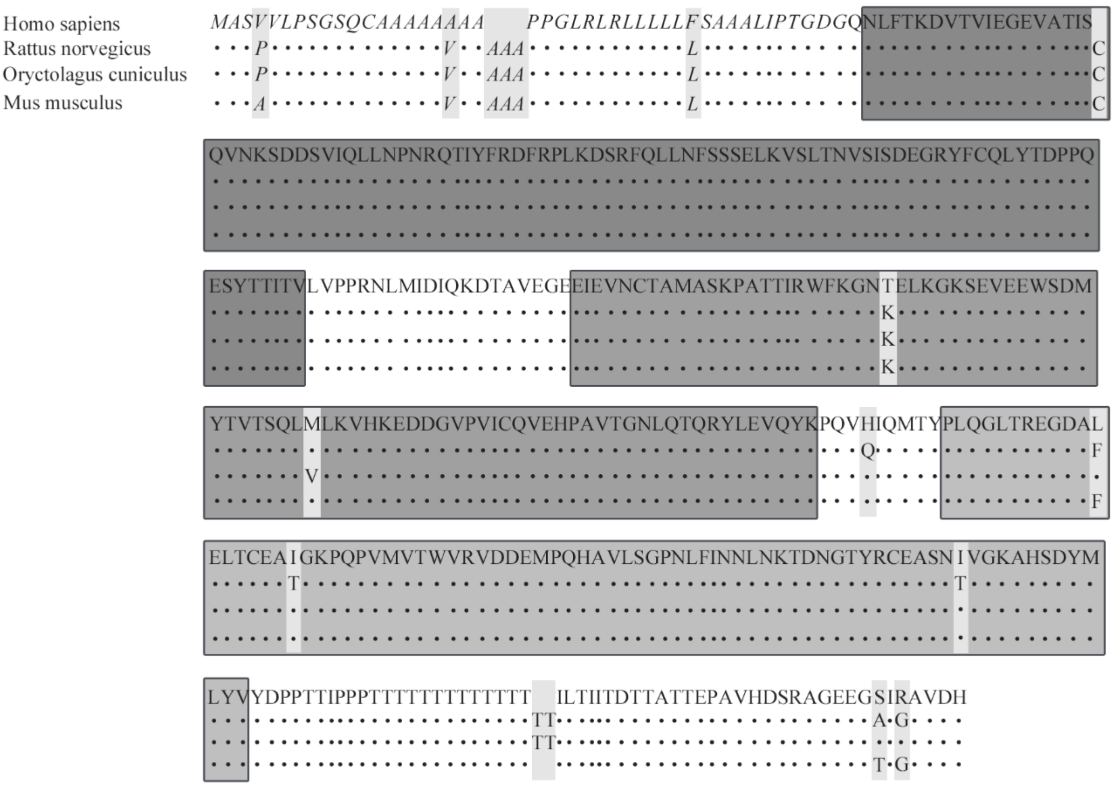

Figure S4. Alignment of the amino acid sequence of the CADM1 ectodomain among humans, rats, rabbits, and mice. The predicted signal peptide is shown in italic. Three Ig-like loops are boxed. Dots indicate amino acids identical to those of the human CADM1. Spaces without letters mean that there are no corresponding amino acid residues in the human CADM1 ectodomain. The positions of residues that are not completely conserved are highlighted by the faint gray background. The GenBank accession numbers are NP_001287973 (Homo sapiens), XP_006243006 (Rattus norvegicus), XP_002708452 (Oryctolagus cuniculus), and NP_997558 (Mus musclus).

Table S1. Cell lines used in the study.

Name	Origin and definition	Doubling time (hour)	Reference
NCI-H441	Human lung papillary adenocarcinoma	58	ATCC[1]
NCI-H522	Human lung adenocarcinoma, stage 2	38	Cellosaurus[2]
RLE-6TN	Rat alveolar type II; SV40-transfected, transformed		
HEC-1B	Human endometrial adenocarcinoma G2	46	JCRB[3]
OMC-2	Human endometrial adenocarcinoma	55-139	Cellosaurus
CNT	Rabbit kidney distal tube; SV40-transfected		
769-P	Human kidney clear cell carcinoma	35	ATCC
Caco-2	Human colon adenocarcinoma	62	ATCC

[1] American Type Culture Collection. https://www.atcc.org/; [2] A knowledge resource on cell lines. https://web.expasy.org/cellosaurus/; [3] Japanese Collection of Research Bioresources. https://cellbank.nibiohn.go.jp/english/.

Table S2. *P*-values by one-way ANOVA and Bonferroni correction.

Cell	ANOVA	Bonferroni				
		Confluence	30	50	70	90
NCI-H441	< 0.001	50	0.0088	–	–	–
		70	< 0.001	0.138	–	–
		90	< 0.001	< 0.001	< 0.001	–
		110	< 0.001	< 0.001	< 0.001	1
RLE-6TN	< 0.001	70		0.157	–	–
		90		< 0.001	0.0069	–
		110		< 0.001	< 0.001	0.0404

Figure 1B: CADM1 expression level.

Cell	ANOVA	Bonferroni		
		Confluence	50	70
NCI-H441	< 0.001	70	0.682	–
		110	< 0.001	0.0025

Figure 1C: Cell height.

Cell	ANOVA	Bonferroni		
		RNA	None	scramble
NCI-H441	< 0.001	scramble	0.360	–
		si-CADM1	< 0.001	< 0.001
RLE-6TN	< 0.001	scramble	0.160	–
		si-CADM1	< 0.001	< 0.001

Figure 3B: CADM1 expression level.

Cell	ANOVA	Bonferroni		
		IgY	U04	9D2
NCI-H441	< 0.001	None	1	< 0.001
		U04	–	< 0.001
NCI-H522	< 0.001	None	0.960	< 0.001
		U04	–	< 0.001
RLE-6TN	0.0013	None	1	0.0041
		U04	–	0.0019
HEC-1-B	< 0.001	None	1	< 0.001
		U04	–	< 0.001
OMC-2	0.0025	None	1	0.0064
		U04	–	0.0042
CNT	< 0.001	None	1	< 0.001
		U04	–	< 0.001
769-P	< 0.001	None	0.0078	< 0.001
		U04	–	< 0.001
Caco-2	0.0059	None	1	0.0156
		U04	–	0.0097

Figure 4B: CADM1 expression level.
